# Exploring Attitudes and Experiences of People With Knee Osteoarthritis Toward a Self-Directed eHealth Intervention to Support Exercise: Qualitative Study

**DOI:** 10.2196/18860

**Published:** 2020-11-26

**Authors:** Rachel K Nelligan, Rana S Hinman, Pek Ling Teo, Kim L Bennell

**Affiliations:** 1 Centre for Health, Exercise and Sports Medicine Department of Physiotherapy, School of Health Sciences The University of Melbourne Parkville Australia

**Keywords:** text messaging, mobile phone, knee osteoarthritis, exercise, qualitative, pain

## Abstract

**Background:**

Knee osteoarthritis (OA) is a highly prevalent and debilitating condition. Exercise is a recommended treatment because of its effectiveness at improving pain and function. However, exercise is underutilized in OA management. Difficulty accessing health care has been identified as a key barrier to exercise uptake. Innovative and scalable methods of delivering exercise treatments to people with knee OA are needed. We developed a self-directed eHealth intervention to enable and encourage exercise participation. The effectiveness of this intervention on pain and function in people with knee OA is being evaluated in a randomized clinical trial.

**Objective:**

This study aimed to explore the attitudes and experiences of people with knee OA who accessed the self-directed eHealth intervention and the features perceived as useful to facilitate self-directed exercise.

**Methods:**

This was a qualitative study embedded within a randomized controlled trial. Individual, semistructured phone interviews were conducted with 16 people with knee OA who had accessed a 24-week eHealth intervention (website and behavior change SMS program) designed to support exercise participation. Interviews were audiorecorded, transcribed verbatim, and thematically analyzed using an inductive approach.

**Results:**

Five themes arose: (1) technology easy to use and follow (website ease of use, SMS ease of use), (2) facilitators to exercise participation (credible OA and exercise information, website features, prescribed exercises simple to do unsupervised, freedom to adapt the exercise to suit needs, influence of other health care experiences), (3) sense of support and accountability (SMS good reminder and prompt, accountable, SMS tone and automation could trigger negative emotions [eg, guilt or shame], inability to contact someone when needed), (4) positive outcomes (knee symptom improvements, confidence to self-manage, encouraged active living), (5) suggestions for real-world application (provided by a health professional preferred, should be provided at subsidized or low out-of-pocket cost).

**Conclusions:**

People with knee OA had mostly positive experiences with and attitudes towards the use of an eHealth intervention that supported exercise participation independent of a health professional. A human connection associated with the eHealth intervention appeared important.

## Introduction

Knee osteoarthritis (OA) is a highly prevalent and debilitating condition [1]. Knee OA clinical guidelines advocate condition-specific information and exercise as first-line treatments [2-6]. Despite this, exercise is underutilized in preference for pharmacological, surgical, or “wait and see” management [7-10]. This is in part due to some patients having difficulty accessing health professionals trained in exercise prescription [11,12], particularly in remote areas [13]. Furthermore, in the absence of health professional input, people with knee OA can lack confidence navigating nonsurgical interventions [14] and motivation to adhere to exercise [11,15]. They may also hold negative exercise beliefs [11,16], which may prevent uptake of and adherence to self-directed exercise. The prevalence of knee OA is forecast to increase substantially due to an aging population and rising obesity. This is predicted to place even greater pressure on access to health care resources [17]. In light of this, innovative and scalable methods of delivering evidence-based, first-line treatments, such as exercise, are needed.

eHealth programs may be one solution to increase exercise participation in people with knee OA [18,19]. While qualitative studies demonstrate that, overall, self-directed, web-based programs designed to support exercise or physical activity are viewed positively by people with OA [20-22], engagement is low [23,24]. This may impede their successful implementation and subsequently their usefulness in facilitating improved health outcomes [25]. Identified facilitators of acceptance and engagement with these programs include content credibility and technology ease of use, while a key barrier is lack of health professional involvement [20,21]. Incorporating health professional input may be one solution to improve acceptance and engagement; however, this does not fully address the problem of health care accessibility.

SMS using mobile phones may be one strategy to improve engagement with OA eHealth self-management programs and support exercise behavior without the need for health professional input. SMS has been shown to effectively increase uptake of healthy behaviors including physical activity, smoking cessation [26], adherence to diabetes self-management, and medication adherence [27]. The combination of a self-directed, web-based intervention supported by SMS has not been evaluated in people with OA.

To explore this, we developed a “light-touch,” self-directed, eHealth intervention that combines a website, “My Knee Exercise,” and a 24-week behavior change SMS program. The effect of this intervention on knee pain and function is currently being evaluated in a randomized controlled trial (RCT) of 206 people with a clinical diagnosis of knee OA [28]. In addition, as patient acceptability is a key component to successful intervention implementation [29], qualitative enquiry is also needed to understand if the eHealth intervention is accepted by people with knee OA to facilitate self-directed exercise. Qualitative enquiry will also inform intervention modifications. The aim of this study was therefore to explore the experiences and attitudes of people with knee OA who accessed the eHealth intervention and identify which features were perceived as useful to facilitate self-directed exercise.

## Methods

### Design

A qualitative study based on an interpretivist paradigm [30] was nested within an RCT [28] evaluating the effectiveness of an eHealth intervention of web-based information and exercise prescription supported by behavior change mobile phone SMS (data collection completed and manuscript in preparation; Australian New Zealand Clinical Trials Registry ACTRN12618001167257). Reporting complies with The Consolidated Criteria for Reporting Qualitative Research checklist [31].

### Participants

Participants in this study were a subsample of those allocated to the intervention arm of the RCT who had completed the 24-week intervention within the past 2 months. Participants were purposively sampled to participate in this qualitative study. Purposive sampling was used to ensure variation across sex, age, geographical location (eg, metropolitan, regional), and responses to 24-week measures of self-reported perceived change in symptoms and of website and SMS usefulness. The sample size was dictated by theoretical saturation, a concept where recruitment ceases when no new information emerges from the data [32]. Ethics approval was obtained from the Human Research Ethics Committee of University of Melbourne (HREC No. 1852367.1). Participants provided informed consent via online consent forms prior to the interview. Initial recruitment for the RCT was from the Australia-wide community via internet sources (social media and online newspapers) and a volunteer database. Eligibility criteria for the RCT included age ≥45 years and a clinical diagnosis of knee OA [5]. Full RCT eligibility criteria are reported elsewhere [28].

Table 1 describes the characteristics of the 16 participants interviewed. The mean age of participants was 63 years, and half (8/16, 50%) were female. Participants lived in locations across all states and territories within Australia, except for the Northern Territory; 9 (9/16, 56%) lived in regional Australia.

**Table 1:** Participant details (n=16).

Pseudonym	Sex	Age (years)	Level of education completed	Employment status	State	Geographical location^a^	Baseline knee pain^b^	Perceived change in knee condition (24 weeks)	Website usefulness^c^ (24 weeks)	SMS usefulness^d^ (24 weeks)
Olivia	F^e^	65	Tertiary	Part-time	NSW^f^	Metropolitan	6	Much better	7	7
Harry	M^g^	73	Tertiary	Retired	Qld^h^	Metropolitan	5	Moderately better	5	6
Charlotte	F	67	Secondary	Retired	WA^i^	Metropolitan	7	Much worse	1	4
James	M	67	Secondary	Retired	VIC^j^	Regional	5	Much better	6	7
William	M	58	Tertiary	Full-time	ACT^k^	Metropolitan	4	Slightly better	2	4
Amelia	F	75	Tertiary	Retired	SA^l^	Metropolitan	5	Slightly better	2	1
Charlie	M	48	Tertiary	Full-time	NSW	Regional	7	Much better	7	7
Liam	M	68	Tertiary	Retired	WA	Regional	5	Much better	6	6
Grace	F	73	Tertiary	Retired	SA	Regional	6	Moderately better	2	4
Joshua	M	62	Secondary	Retired	Qld	Regional	4	Much better	5	5
George	M	56	Secondary	Full-time	NSW	Regional	5	Moderately better	5	6
Lucy	F	59	Secondary	Part-time	NSW	Metropolitan	6	Moderately better	5	4
Oliver	M	53	Secondary	Full-time	WA	Regional	7	Slightly worse	5	2
Sophie	F	57	Tertiary	Part-time	SA	Metropolitan	6	Much better	5	6
Emily	F	55	Tertiary	Part-time	Tas^m^	Regional	7	Much better	6	6
Chloe	F	65	Secondary	Retired	WA	Regional	8	Slightly better	6	6

^a^Defined according to The Australian Statistical Geography Standard Remoteness Structure [33].

^b^Self-reported overall knee pain in the past week rated on a numeric rating scale, ranging from 0 to 10, where lower scores indicate less pain.

^c^Agreement with the statement “I thought the website I accessed as part of the study was useful in helping me manage my painful knee,” rated on scale ranging from 1 to 7 (1= strongly disagree; 7=strongly agree).

^d^Agreement with the statement “I thought the mobile phone text messages I received were useful in helping me manage my painful knee,” rated on a scale ranging from 1 to 7 (1= strongly disagree; 7=strongly agree).

^e^F: female.

^f^NSW: New South Wales.

^g^M: male.

^h^Qld: Queensland.

^i^WA: Western Australia.

^j^Vic: Victoria.

^k^ACT: Australian Capital Territory.

^l^SA: South Australia.

^m^Tas: Tasmania.

### Intervention

**Figure 1:**
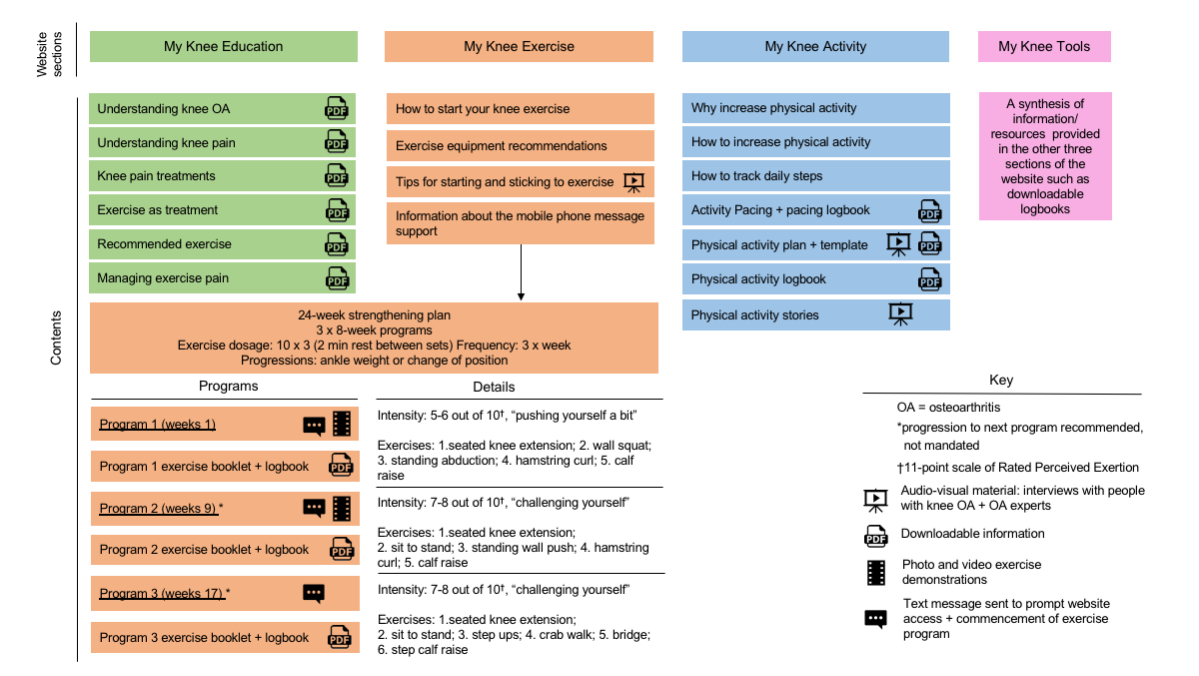
Description of the content in the 4 sections of the intervention website, "My Knee Exercise." OA: osteoarthritis.

Full details of the digital intervention are described elsewhere [28]. In summary, the intervention included a website, “My Knee Exercise,” and a 24-week mobile phone SMS behavior change program. The website contained information about knee OA, exercise, and general physical activity and prescribed a 24-week lower limb strengthening program to be completed 3 times per week. Figure 1 outlines the contents of the website. The website was developed by the researchers (RN, KB, RH), and 3 people with knee OA provided feedback on a prototype, which informed the final design. The strengthening exercises were based on those found to be effective at reducing pain and improving physical function in people with knee OA (when prescribed by a physiotherapist) in our prior clinical trials and were originally developed by the researchers, who are physiotherapists, in collaboration with a clinical physiotherapist [34-37]. Exercises focused on the hip, knee, and ankle such as sit-to-stand, seated knee extension, and calf raise. Exercise instructions were provided in text and visual formats (photo, video). Exercise equipment (eg, ankle weights) was recommended to progress the exercises, and information about where these could be purchased was provided. Exercise instructions and logbooks were available to download. Participants could access the website whenever they chose.

**Figure 2:**
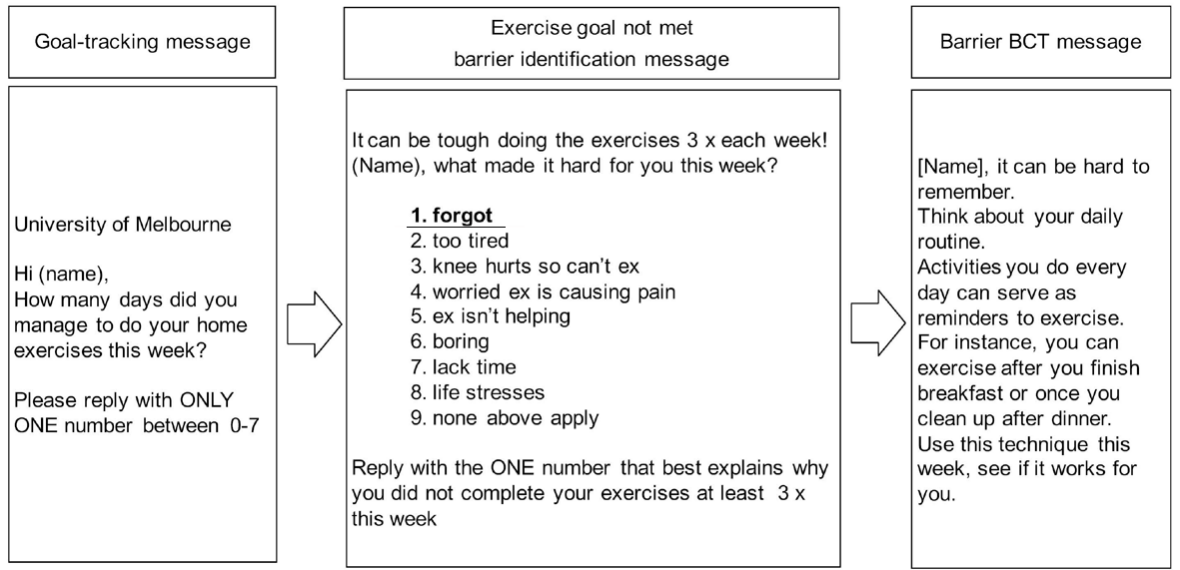
Example automated message sequence for a person with low exercise adherence (reporting <3 exercise sessions over the past week) and reporting their main barrier to exercise as "forgot." BCT: behavior change technique. [38] Reproduced under the terms of Creative Commons Attribution 4.0 license.

The 3-times weekly strengthening exercise program was supported by an automated SMS program. The SMS program was rigorously developed using behavior change theory [38], with input into message tone and wording provided by 12 people (7 academics working in knee OA, 4 clinical physiotherapists, and 1 person with knee OA) in addition to the researchers who developed the program (RN, KB, RH, and a behavior change expert). In brief, the program functioned by prompting participants, on Mondays, to self-report how many strengthening exercise sessions they had completed in the previous week. Participants then received an SMS response based on their reported level of adherence. Adherent participants (≥3 exercise sessions/week) received a positive reinforcement SMS. Low-adherent participants (≤2 exercise sessions/week) received an SMS asking them to choose, from a prespecified list of exercise barriers, what made it challenging to complete their exercises 3 times as recommended. This triggered a response SMS containing a behavior change technique suggestion related to the selected barrier (see example in Figure 2). Irrespective of self-reported adherence, participants also received SMS (initially twice weekly and reducing to once a fortnight by week 24) containing behavior change technique suggestions to motivate and facilitate exercise participation. Participant responses not recognized by the program (eg, responses not using suggested keywords) triggered a “response not supported” message encouraging them to try again or contact program staff if needed. Participants received on average 2-5 SMS per week, dependent on weekly responses, with the frequency of contact declining over 24 weeks.

After randomization, participants in the intervention received a welcome email that provided website access (a URL and individualized username and password), asking them to login to the website within 7 days and were scheduled to receive their first SMS, after a period of at least 5 days beginning with Monday. The first SMS in the sequence was a prompt to self-report the number of prescribed exercise sessions completed in the previous week.

### Interviews

Individual semistructured telephone interviews were conducted by RN (RCT coordinator, PhD candidate, and physiotherapist), who was also involved in intervention design and responsible for recruitment for the RCT. The interview guide was developed by the authors (RN, KB, RH) and aimed to explore experiences overall and with individual elements of the eHealth intervention (Multimedia Appendix 1). All interviews were audiorecorded, transcribed verbatim by an external service, and stored and de-identified in a password-protected, secure computer file on the university server. Pseudonyms were assigned to each participant to maintain confidentiality.

### Data Analysis

An inductive thematic analytical approach was applied using the 6 phases outlined by Braun and Clarke [39,40]. Data were coded by 2 independent researchers, one who conducted the interviews (RN) and the second (PT) who had no prior involvement in design or evaluation of the eHealth intervention. Full details are provided in Table 2. Analysis occurred iteratively and reflectively with forward and backward movement within Phases 1-3. To present the data, pseudonyms were assigned to exemplary quotes.

**Table 2:** Thematic analysis process conducted based on the phases described by Braun and Clarke [39].

Phase	Description of the process
1. Familiarizing yourself with your data	Data were transcribed by an external company. All transcripts were read by RN for accuracy and to note initial ideas.
2. Generating initial codes	Two researchers experienced in qualitative analysis (RN, PT) independently coded all transcripts and collated data relevant to each code. The 2 researchers met after coding transcripts, in blocks of 4, to discuss and seek agreement of codes and their meaning before proceeding to the next 4 transcripts.
3. Searching for themes	RN and PT independently grouped codes into potential subthemes and themes, gathering all data relevant to each potential theme. They then met to compare, discuss, and seek agreement on themes. Agreement between the 2 researchers was strong; therefore, a third coder was not required.
4. Reviewing themes	KB read all transcripts. RN, PT, and KB checked that subthemes and themes truly represented the coded extracts and the entire data set.
5. Defining and naming themes	All authors discussed and refined subthemes and themes as well as definitions and names for each.
6. Producing the report	RN developed the draft of this manuscript. All authors provided input and approved the final version.

## Results

### Thematic Analysis

From the data, 5 themes were identified. Multimedia Appendix 2 outlines the themes, subthemes, and supporting quotes.

#### Theme 1: Technology Easy to Use and Follow

For the first subtheme of “website ease of use,” participants found the website easy to get around, the navigation easy to follow, and the information provided easy to grasp regardless of technical abilities:

I’m not the smartest computer user in the world, but if I can do it, I reckon anybody can do it.

George

For the second subtheme of “SMS ease of use,” participants mostly found the SMS program easy to use: “It's just simple” [Olivia]. However, one participant reported difficulty replying to messages in the recommended format:

Sometimes with the SMS, I'd put the letters and the things around the wrong way… it was very particular, you know, you had to do it in the right…But other than that, no worries at all.

Lucy

#### Theme 2: Facilitators to Exercise Participation

The first subtheme was “credible OA and exercise information.” For many, the fact that the intervention was developed and delivered by a university, a credible source, was appreciated and gave them confidence to apply the eHealth program’s information and recommendations. Participants felt they could trust the information, which reinforced and improved their understanding of OA and the role of exercise in managing their knee symptoms. This built their confidence to exercise despite discomfort and without health professional guidance.

The second subtheme was “website features.” Participants appreciated certain features of the intervention that enabled exercise participation. Participants felt the website was comprehensive, and the information in the “My Knee Education” section of the website was easy to understand and helpful in supporting self-directed exercise being “more than what the doctor has given you” [Emily]. They found that the written exercise instructions, exercise pictures, and videos helped them master the exercises easily without needing supervision. Some participants valued being able to access the website frequently to view the exercises, while others only accessed the website once or twice, preferring to download and print exercise sheets and logbooks.

The third subtheme was “prescribed exercises simple to do unsupervised.” Participants appreciated the simplicity of the recommended exercises. As they perceived the exercises as simple, they believed they did not need supervision:

An allied health (person) to actually monitor the exercises was not necessary.

Harry

The fourth subtheme was the “freedom to adapt the exercise to suit needs.” Participants reported the freedom to use a flexible approach to execute and progress their exercise program over the 24 weeks to suit their needs. As a result, how each participant completed the recommended exercise regime varied greatly. For example, some completed all prescribed exercises at least 3 times a week, many chose not to add additional weight, and others replicated the exercises by doing similar activities in their daily routines. Some also chose their own exercise or physical activity program to complete at the recommended frequency, typically because they found the prescribed exercises too easy and boring. In addition, most participants expressed difficulty with at least one prescribed exercise (the wall squat most frequently reported). This, however, did not deter participants from completing the unsupervised program. If a recommended exercise caused pain, this was typically managed by leaving out the specific exercise and continuing with the remaining exercises or substituting with their own exercise.

The fifth subtheme was the “influence of other health care experiences.” Many discussed their previous experiences with health professionals that influenced their willingness to undertake unsupervised exercise. This included already being familiar with exercise prescription due to prior health professional input and dissatisfaction with past face-to-face care received.

#### Theme 3: Sense of Support and Accountability

The first subtheme was “SMS good reminder and prompt.” Participants felt the text messages were a good reminder and prompted them to continue exercising. Most participants appreciated the predictability of the messages, receiving them at the same time each week. This encouraged them to complete their exercise in anticipation of having to report their weekly exercise sessions each Monday.

The second subtheme was “accountable.” Many of the participants reflected that the SMS program supported their weekly exercise by keeping them accountable to the research team or their commitment to the exercise program. Many described that the messages felt like someone was checking up on them:

You felt like you had to do it because you were going to get checked up…

Chloe

It was like a devil sitting on my shoulder going “have you done your exercises?” Oh, my God, I can only put two in for an answer this week; I’ve got to do better next week.

Sophie

The third subtheme was “SMS tone and automation could trigger negative emotions (eg, guilt/shame).” Participants frequently described feelings of guilt or shame when receiving an SMS particularly if they had not completed the recommended exercise frequency. Most believed this facilitated exercise participation. A few participants did, however, find the message response to exercise low-adherence demotivating: “it was a reminder of the bleeding obvious” [Amelia]. The automated and unsupervised nature of the SMS program was problematic for some, especially in extenuating circumstances:

When I got the planter fasciitis and the texts were coming through…they just kept coming, and it was kind of like a little shame thing.

Charlotte

The fourth subtheme was “inability to contact someone when needed.” Many participants thought it would be beneficial if the SMS program allowed them to provide more detail or converse with someone to better explain low adherence, particularly when the reason did not relate to the options provided. Some also wanted the ability to ask a question or contact someone about the exercise program or their condition if needed.

#### Theme 4: Positive Outcomes

The first subtheme was “knee symptom improvements.” All participants felt the 24-week intervention had benefited them in some way. Many reported reductions in knee pain. This enabled them to walk more, rely less on pain medication, and delay or avoid knee surgery.

The second subtheme was “confidence to self-manage.” Most participants expressed improved confidence in their ability to self-manage their condition. This included greater confidence to maintain their preferred lifestyle. For example:

…it’s helped me not only with my mobility but my self-confidence to be able to go, yeah, I can get up there all right and come down there.

Grace

Several participants also reported the program helped motivate them to lose weight:

Not only has it changed the strength in my knees and reduced the amount of ongoing pain that I have with them, it’s also inspired me to lose weight. I’ve actually lost at this stage — about 14 and a half kilos.

George

The third subtheme was “encouraged active living.” Participants reported increases in physical activity and greater enjoyment in being physically active, which they attributed to their participation in the study. Except for one participant, all expressed a desire to continue to exercise and be physically active to maintain improvements in their condition.

#### Theme 5: Suggestions for Real-World Application

The first subtheme was “provided by a health professional preferred.” All participants would recommend the program beyond the research environment. Relating to how participants could see the intervention being used outside the research environment, most suggested it could be provided by a health professional, particularly a general practitioner or a physiotherapist, to enhance or improve care. Some also believed promoting the intervention through social media was suitable.

The second subtheme was “should be provided at subsidized or low out-of-pocket cost.” Most participants believed costs of participating in the intervention should be subsidized by private health insurers or government initiatives. A few felt strongly that themselves, the user, should pay “so long as it wasn’t too expensive” [Grace], perceiving that this might support adherence.

## Discussion

### Principal Findings

This study explored the experiences and views of people with knee OA who participated in a self-directed, 24-week eHealth intervention designed to facilitate exercise participation. Overall, participants described positive experiences, valuing the simplicity and comprehensiveness of the resources (technical and content) and the regular SMS messages, both of which supported self-directed exercise. However, the SMS automation and tone were problematic for some people when weekly exercise was not completed. Human connection associated with the eHealth intervention also appeared important.

Our findings highlight that simple-to-use technology that conveys easy-to-understand information is well received by people with knee OA. This is in accordance with the findings of a qualitative study that investigated the preferences of people with chronic joint pain regarding the development of a web-based version of a face-to-face self-management program [20]. In order to engage with a web-based version of the program, participants believed it should be easy to understand and navigate, as well as be free from jargon. Participants also valued the eHealth intervention being developed and delivered by a credible source, a university. This influenced their confidence in and acceptability of the self-directed exercise program. Trustworthiness is a similar finding in other OA studies investigating eHealth interventions [20,21]. Furthermore, participants in our study believed the intervention should be provided by health professionals. Endorsement from a trusted health professional is a key facilitator to the adoption of eHealth programs [25]. Therefore, to facilitate implementation of the eHealth intervention, future studies could explore how health professionals might take an active role in dissemination.

In our study, participants valued the SMS messages as a predictable exercise prompt. However, for some people, the automation and tone of the messages were demotivating or evoked feelings of guilt when weekly exercise was not possible (particularly for reasons beyond their control such as an unrelated health problem). To our knowledge, there are no other studies exploring the views of people with knee OA toward the use of text messages to support exercise participation. People with diabetes who received twice weekly text messages to support physical activity participation valued the text messages as a functional reminder but many disliked receiving repetitive messages, describing them as “nagging” [41]. Another study explored the use of text messages to promote exercise in older adults and found participants valued the messages as encouraging and an important “push” to exercise [42]. However, similar to our findings, the messages could cause feelings of guilt when exercise was not completed. Overall, text messaging appears to be an accepted and valued way to remind or prompt people to complete regular exercise. However, such programs may also simultaneously evoke negative emotions. Possible strategies to address this issue, as described by participants, could include revising the tone and tenor of these messages and reducing the frequency of messages sent to people who are unable to exercise due to unrelated health concerns or personal circumstances.

Although the autonomy afforded by the eHealth intervention was valued, our findings also suggest a human connection was important to participants. Most participants felt that the regular SMS made them feel accountable to the people behind the program or research team, which facilitated regular exercise. Commitment to study researchers has been identified in other qualitative evaluations of digital OA self-management programs [21,43]. This has implications for translation into real-world settings, as the perception that someone is “behind” the digital program appears vital. In addition, participants in our study suggested the intervention could be improved by having the ability to contact someone when needed, for example, to further explain reasons for low adherence or ask a clinical question. When using digital self-management programs, people with knee OA tend to prefer some form of support or therapist interaction [20]. Other studies have evaluated blended interventions where digital knee OA programs are supported by therapist input. One study explored the experiences of people with knee OA who had completed a 12-week digital physical activity program supported by up to 5 face-to-face physical therapy sessions [43]. Some participants described the physical therapist’s involvement as positive, tailoring the digital program and monitoring their progress, while others felt it was restricting, particularly when the therapist did not know how the digital program worked. Another qualitative study evaluated the experiences of people with hip or knee OA who had participated in 6 weeks of “Joint Academy,” a digital education and exercise program supported by 1:1 online written contact with a physical therapist [22]. Some participants in this study valued the interaction with their therapist, especially if they experienced pain during an exercise, while others felt the contact was unsatisfactory and that feedback and encouragement on their performance was lacking. These results, in combination with ours, may indicate that people have different preferences for the level of support that should be provided with eHealth self-management programs and highlights that a one-size -fits-all approach to implementation may be inappropriate. As the integration of eHealth interventions into usual care facilitates their successful implementation [25], one solution may be to integrate the eHealth intervention into a stepped model of OA care [44,45], where it is provided as the first “step” in the care plan. Stepped care is where support or interventions are provided in “steps,” with input escalating based on an individual’s outcomes or preferences for care [46]. This may better ensure the right level of input is provided to meet an individual’s needs.

### Strengths and Limitations

Our study had several strengths and limitations. Strengths include that this study was nested within an RCT, allowing for the comprehensive and robust evaluation of the eHealth intervention; use of purposive sampling to ensure a variety of participants was included (age, sex, geographical location, education level, and 24-week outcomes of self-reported perceived improvements and resource usefulness); conduct of all interviews within 2 months of completing the study to facilitate accurate recall; and independent data coding by 2 researchers (RN, PT) to formulate themes, which were reviewed for accuracy and completeness by a third researcher (KB) who also read all transcripts. There were several limitations. There was potential for bias as participants self-selected to volunteer for the overarching RCT advertised as investigating different electronic and digital resources to support knee OA management. Therefore, participants may have held more favorable views regarding the use of technology at the outset. Selection bias may also have occurred in the recruitment for this qualitative study, despite the use of purposive sampling, as participants with more favorable opinions of the eHealth intervention may have been more inclined to consent to be interviewed. However, we attempted to overcome this by deliberately recruiting people with low self-reported perceived usefulness of the resources and low self-reported perceived overall improvements in their knee condition (see Table 2). The researcher’s (RN) perspectives and potential prior relationship with participants could have affected findings, as she was the interviewer for this study, the participant recruiter for the RCT this study is embedded within, and involved in developing the digital resources. We attempted to address this by selecting a second coder for analysis who had no prior involvement in the design or evaluation of the resources.

### Conclusions

In summary, we found that people with knee OA had mostly positive experiences and perspectives of the eHealth intervention. Overall, participants valued the simplicity and comprehensiveness of the resources (technical and content) and the regular SMS prompts, which supported participation in self-directed exercise. Our findings demonstrate the intervention may be an acceptable resource for people with knee OA to encourage self-directed exercise participation. However, a human presence associated with the intervention appears important. Future modifications to the intervention could include adaptations to parts of the SMS program (tone and automation) to minimize inciting negative emotions when exercise is not possible. Further research should explore the real-world application of the intervention, including how the intervention could provide more personalized support for individuals wanting greater input and how it could be integrated into OA care or health professional consultations.

## Appendix

Multimedia Appendix 1 Semi-structured interview guide.

Multimedia Appendix 2 Themes, sub-themes, and exemplary quotes. Pseudonyms used for participant names.

## Abbreviations

OA: osteoarthritis
RCT: randomized controlled trial
