# Supplementary material for: Exploring Attitudes and Experiences of People With Knee Osteoarthritis Toward a Self-Directed eHealth Intervention to Support Exercise: Qualitative Study
Source: JMIR Rehabil Assist Technol. 2020 Nov 26;7(2):e18860. doi: 10.2196/18860 (PMC7728537; doi:10.2196/18860)
Supplement: Multimedia Appendix 1 [file rehab_v7i2e18860_app1.docx]

Multimedia Appendix 1. Semi-structured interview guide

1. Can you tell me about your overall experience with the website and SMS over the last 6 months?

Prompts: What did you like? What didn’t you like? What changed, if anything, for you because of using the website/SMS?

The next few questions relate to each section of the website.

1. Thinking about the **My Knee Education** section. This was the section that contained information about knee OA, knee pain and exercising with knee OA.

How useful was the information in this section for you in managing your knee problems?

Prompts: What did you find useful? What was not useful? Was there other information you would have liked to see included? How did you use the information to change the way you manage your knee problems? What key points do you remember from the information in this section? Thoughts on videos?

1. Thinking about the **My Knee Strength** section now. This section of the website prescribed a strength exercise program to be completed 3 times each week.

How did you go doing the recommended strength exercise program over the last 6 months?

Prompts: What information was most useful? Least useful? Is there any other information you would have liked to see?

How did you go doing them three times each week for 6 months?

What helped you stick to the exercises? What made it hard for you? What would have helped you more?

How did you go progressing your program over the 6 months? Were there any challenges you faced?

1. How did you feel doing the strengthening exercises on your own without involvement from a healthcare provider like a Physio?

Prompts: how confident were you performing the exercises correctly? how motivated were you to do the exercises on your own at home? Did you encounter any problems and if so, how did you try to overcome them?

1. What are your thoughts on the recommendation to buy ankle weights to progress your strength exercises?

Prompts: did you purchase any exercise equipment? Why/why not?

1. Thinking about the **My Knee Activity** section now. This section provided information about increasing your general physical activity levels.

In the last 6 months how did you go increasing your general physical activity?

Prompts: Why/why not? What types of physical activity did you do? Was the information in this section useful/not useful for you to increase physical activity levels? What information was most useful? Least useful? Is there any other information you would have liked to see?

What helped you? What would have helped you more?  How did you feel increasing physical activity without direction from a healthcare professional like a physio?

1. Finally thinking about the **My Knee Tools** section of the website. This section contained a range of resources used within the website, such as the logbooks. How did you use the **My Knee Tools** section of the website?

Prompts: What was useful? What was not useful? How could this section have been more useful for you?

1. Now thinking about the mobile phone messages. These were designed to support you to do your strengthening exercise 3 times each week.

How useful were they in helping you stick to your strengthening exercises 3 times each week?

Prompts: In what way were they useful? In what way were they not useful for you?

How relevant were the messages to you and your personal situation?

What are your thoughts on the message frequency? What are your thoughts on the content of the actual messages?

How could the mobile phone messages have been more useful to help you stick to your strengthening exercises 3 times each week over the 6-months?

1. Thinking about your doctor, and other healthcare providers that you may have worked with, your friends and family……. what did they think about you using the website and SMS to manage your knee problems?

Prompt: In what ways did they support you? In what ways didn’t they? Did their opinions influence how you used the website or SMS program to manage your knee problems?

1. Would you recommend the website and the SMS program to others?

Prompts: Consider both the website and mobile phone messages. Why/why not? Will you continue using anything you have learned from the program in the future? Why/why not?

1. Is there anything else you would like to add?
