# Supplementary material for: Exploring Attitudes and Experiences of People With Knee Osteoarthritis Toward a Self-Directed eHealth Intervention to Support Exercise: Qualitative Study
Source: JMIR Rehabil Assist Technol. 2020 Nov 26;7(2):e18860. doi: 10.2196/18860 (PMC7728537; doi:10.2196/18860)
Supplement: Multimedia Appendix 2 [file rehab_v7i2e18860_app2.docx]

| **Multimedia Appendix 2. Themes, sub-themes, and exemplary quotes. Pseudonyms used for participant names.** | |
| --- | --- |
| **Sub-theme** | **Exemplary quote** |
| **Theme 1: Technology easy to use and follow** | |
| Website ease of use | (Lucy) “I found the website was easy to get around, and I'm not a very technical person, so I found it was pretty good. So yeah, no problems at all.”  (James) “It’s easy to grasp, it’s easy to follow.”  (Olivia) “It was pretty basic, you know, the way you could watch the video and then you can take a bit at a time, so you could understand and then you could do it from one exercise to the next without having to chase it up. So, it was pretty easy to follow. And again, I’m not the smartest computer user in the world, but if I can do it I reckon anybody can do it.” |
| SMS ease of use | (Olivia) “It's just simple”  (William) “The way the numbering and the lettering was done made it all, I think, easy to understand for me.”  (Lucy) “Sometimes with the SMS's I'd put the letters and the things around the wrong way… it was very particular, you know, you had to do it in the right…But other than that, no worries at all” |
| **Theme 2: Facilitators to exercise participation** | |
| Credible OA and exercise information | (Charlotte) “I figured it was a study that you had done through university... I figured it was a good study that you would have researched a lot of things, and I thought it was worth a try, you know, like definitely worth a try…I always thought that, because my mother had three knee replacements, and people would say oh, it’s bone on bone. But when I read your thing, it was saying that’s not the case, and that moving your knee is the best thing for it. So that was really interesting, So, I did get some good information off it, I really did”  (Olivia) “It was good, because up until I read the first part of that introduction, basically, to the whole process, when I used to start exercising my knees got worse... But what it’s done is it’s given me the information to say that, yeah, you’ll suffer some initial probably deterioration or increase in knee pain, but if you’re consistent and you keep going, that will then strengthen the knees and help reduce that pain, which is exactly what’s happened.”  (Emily) “I think probably the biggest thing that worked for me…Well, what I thought initially was if it hurts, don’t do it. So, changing my idea was what I needed, yeah.” |
| Website features | (Olivia) “The website is good. It shows you how to do the exercise and there's also a video to watch on how to do it - so that really helps… I haven't seen the physio after this one - actually saved me plenty of money going to the physio, because care plan is only five, you can only get five care plan - usually the physio charge us more than the care plan. So, with this video and exercises that is suggested it actually saved me plenty of money. And I haven't seen the physio as I've told you, but I was able to mention this to my GP and he was very happy about this.”  (James) “And the good thing was the pictures and that shows you how to do it, and where you could do it; against the table, against the bench, that sort of stuff. So, it was very, very good in the sense that it showed you how to do all the exercises.”  (Amelia) “So I found the videos … the descriptions were good and clear, but the combination of the textual descriptions and the videos helped me just master it very quickly, and they were simple ones. Very simple.”  (Charlotte) “So printing them off and just having them printed there in front of you, you can just do them. You can do one more, and you just turn it over and you’ve got the page with the next lot there in front of you instantly. So, I found that helpful.”  (Sophie) “The website, I used initially to download the logbook and the pictures of the exercises, and then I didn’t really go back to it at all, because I was on the SMS train.” |
| Prescribed exercises simple to do unsupervised | (Harry) “The exercises were easy, the exercises, I thought, were relevant and they were logical and so far as I was concerned, they worked. So, having a physio to monitor, I don’t know what the physio would have done, but having a physio or an Allied Health to actually monitor the exercises was not necessary.”  (Charlotte) “I didn't mind doing the exercises on my own. I had the sheets, you have the things, so that was okay; .... I mean, you’re sitting in a chair and you’re lifting your leg up, and you are sliding down a wall. It’s not super difficult to follow them. I don't know how wrong you could get them.”  (Amelia) “The reason that I felt ok with it was that they were simple enough that I didn’t need to have somebody saying “No, you need to be standing there” or “You need to be doing this” to get a good result. I didn’t ever feel unsafe”  (Sophie) “They were easy. The exercises weren’t ridiculous. They were easy to do, easy to increase. I bought the ankle weight thingies to help me. They were simple, and easy to understand.” |
| Freedom to adapt the exercise to suit needs | (Amelia) “Sometimes I did them in a batch as if they were an exercise cycle of however long it took, and sometimes I fitted bits and pieces in around other things that were happening in my life.”  (Emily) “I’d do it standing at the breakfast bar. So, I’ve been mindful of when just standing up there doing a bit of cooking or something like that, I can fit this in while I'm looking at the TV as well. So, it wasn’t a big ask.”  (Harry) “I actually started off by getting some old socks and I filled them, I put some sand in them and I used them.”  (Lucy) “One of my favourites was the standing side leg one – I love the hamstring curl and the calf raises. I actually added bands to those at different times and an ankle weight and things like that, to extend them further in the second part…I tightened the band, I made it a smaller band, rather than a larger band, to get more resistance on it, and added extra weight in the ankle weights.”  (Michael) “I just did what I could when I could, whether it was the right exercises I don’t know. Well, if I was sitting at the desk doing paperwork I might try and do something there. And if not, I was doing it when I was out walking around because I sort of work a 12-hour day. I just did what I can when I can whether it was days or nights because I work shift work sometimes.”  (Liam) “You didn’t have to sit down and do the regime every day, as long as you did something that replicated that regime. It was much simpler. And if I’m climbing up and down a ladder painting a wall, putting a roof on or something all day, it’s doing a lot – the exercise counted, what you’re requesting there. A lot of those things are targeted for people that are like, I can’t walk anymore and I’m going to sit down and watch television all day. Whereas I don’t do that.”  (Chloe) “I didn’t mind actually because you can do them any time of the day that you want. If you feel a bit wonky or something you can just take it easy and then you just push yourself a little bit and sometimes if you push yourself a little bit too hard, they’ll say, calm down a bit, and I’m one for pushing yourself just that little bit harder because I think it really helps. So, I think when you do them on your own, you can do it to your own, to your own time.”  (Harry) “The exercise that I had trouble with, the leg stretches, I just did the basic number and that was it. So those ones I didn’t try and stretch myself to do anything more and I just said okay, they're in the programme, I'll just do them and that’s it. I'm not going to do anything more, so they just became a basic. But, certainly, I didn’t need any kind of Allied Health professional to try and work through those. They weren’t needed.”  (William) “Well, I thought at the time when I was looking at it a lot of them were quite basic, leg lifts and sitting squats and those sorts of minor knee activity. I thought I don’t need to do that because I’m relatively strong anyway, and I do a lot of cycling, and so I thought if I just concentrate on my cycling I probably don’t need to do these other exercises. So, my whole focus around knee strength, my knee strength, was around cycling and within a certain range of motion, no standing or no very heavy pedalling. And so, I guess I didn’t do a lot of those other exercises that were presented.” |
| Influence of other healthcare experiences | (James) “I had seen a physiotherapist before, but I had very little information on what I could do. It seemed to be like, you know, it’s your age, bad luck, that sort of thing.”  (Olivia) “With the physio sometimes you stay there for half an hour, or forty-five minutes - it's not as comprehensive. They will just give you general information. And most physio, because I have been to a few physio already, they would manage two or three patients in one session. So, let's say for example you do this one exercise, and they say: ok you do that one - and then they will leave you. You do that for ten minutes, and then they would attend to the other patient. For this one nobody monitors you but yourself and the video really helps.”  (Lucy) “I've worked with exercise physiologists before so I kind of knew – I knew how to do all the exercises that were in the programme…I kind of liked it better because it used to bug the crap out of me when I had to go into the exercise physiologist, and you sort of stand there and they'll come and they'll watch you and say: ok no worries – and they walk away and they go to somebody else…. And it kind of, I don't know – having worked with health practitioners before I know what the system is like and I'm much better on my own doing exercises. I much prefer to do it on my own.”  (Grace) “No, it wasn’t a problem because I have, in the past, been a gym goer, so understanding how to apply an exercise wasn’t actually a problem, I got the gist of that very easily, so, no, that wasn’t a problem.” |
| **Theme 3: Sense of support and accountability** | |
| SMS good reminder and prompt | (Olivia) “The good thing about the SMS is that it reminds you what to do, it's a good reminder.”  (Charlotte) “They were great. When you are capable of doing the exercises, they’re fantastic. It’s a really good reminder.”  (Amelia) “Every now and then I’d think “Oh, Monday is coming up. How many times have I done it this week? Oh, I better do another one.”  (Sophie) “They were useful in reminding me that I needed to do my exercises. They were annoying because they reminded me that I maybe hadn’t done them last week, hence my love/hate relationship with them. But, yes, I probably wouldn’t have stuck to it without them.” |
| Accountable | (George) “So those knee exercises were giving me the drive, and it was the SMS messages that were contributing to that too because I liked to be honest when I was sending the messages back so I made sure that I complied all the time.”  (Charlie) “I think for me it’s about being accountable. It wasn’t so much about the messages, it was about the transfer of information for what I had done.”  (Lucy) “Like I said, it just made me – how can I put it? Don't be so lazy some days, you know. I was like: get off your bum and go and do it. You know what I mean? I suppose that's a change. And the fact that I had to do it, the fact that I had made a commitment to do it. I wasn't going to let myself down or the programme.”  (Emily) “You get a text halfway through the week, and it says you're doing a good job or whatever, or the one after and that. I think it’s that someone constantly niggling at you just going – Someone there’s, you know.”  (Sophie) “It was like a devil sitting on my shoulder going ‘have you done your exercises?’ Oh, my God, I can only put two in for an answer this week, I’ve got to do better next week.”  (Chloe) “I felt like someone was checking up on me. You felt like you had to do it because you were going to get checked up during the week so you needed to do your bit so that you had something to send back in the message.” |
| SMS tone and automation could trigger negative emotions (e.g. guilt/shame) | (Charlotte) “When I got the planters fasciitis and the texts were coming through, it made me feel really guilty, and I would confess, when I tried phoning you or email, and then they just kept coming, it was kind of like a little shame thing...I wasn’t able to do them, it doesn't accept it and then the thing comes, you get the return message with just try more next week. You’re just thinking, like didn't they understand?”  (Amelia) “The minute I get an automated response a half a minute after sending something, I’m immediately demotivated, I’m immediately distanced…If somebody doesn’t do a round of exercises they’ve agreed to, they don’t need to be told doing them is important  [laughs]…it was a reminder of the bleeding obvious” |
| Inability to contact someone when needed | (Charlotte) “…the messages were good. You just need that tweaking, that if something happens, the person can leave a message or text you back or something, if there’s a reason. You know, if there’s a reason that they’re not doing it.”  (James) “but, yeah, as to why, you know, why maybe it could be handy, if you couldn’t have done the three, why? What happened? Is there any other reason? You might’ve been away, had a sick child or whatever, you just couldn’t get around to doing it…you have got someone to follow up if you need to, like someone like you, really follow it up if there’s a real problem.”  (George) “It’d be nice if you had the opportunity to, when you respond to those emails [SMS], if you could just ask a short question….I like the encouragement it gives you to keep going.  You know, your knee pain’s getting better and the response is a good job. But again, I think people would find it, you know, if they could ask a question. You know, put their 3C or 4B or whatever they’re doing, and then go, hey, I’m feeling this, or I want to do this, or I’m struggling with that.” |
| **Theme 4: Positive outcomes** | |
| Knee symptom improvements | (Olivia) “It has improved so much. I think if you do it on a regular basis, it's recommended at least three times a week, it does strengthen your knee and the pain is gone I think, after a couple of months, doing regular exercise. I don't know if I told you, there was a time before I started this one that I cannot even walk. I was on the street and it was really so painful that my brother had to pick me up from one corner of a place where I was. And I cannot even step on my foot, for the knee, and he has to hire a wheelchair for me.”  (Harry) “I'm hoping that something like this is going to delay the need for surgery. I think it’s done that. Personally, I don’t think it could have done any more than it’s done”  (James) “Just probably over time the fact that I could feel there’s no pain there, it was getting rid of the pain, and I wasn’t getting as much pain. I wasn’t needing to put the strap on or the bandage on it, the elastic bandage on it, and just to say “oh geez, it is working”. And the fact that I hadn’t been taking Panadol Osteo, great! Not that they don’t work that well, but I was taking them because of the pain in my knee.”  (Charlie) “As I said, my knee’s completely healed and I didn’t think it would ever be the way it is now. So, for two years I was putting up with pain and hobbling around, and now I’m riding pushbikes and bush walks and all sorts of groovy things.”  (Sophie) “It’s not as puffy; it’s definitely not as painful during the day, and I’m not having the discomfort at night at all, so it’s been really good. So, make sure you let me know when you’ve refined your resources so I can go back and use them again, please.”  (Emily) “It’s worked for me. It has a lot more information than what you’d get from your GP. It’s a motivational experience where you can get exercises and learn something new, and how to give your knee strength. The text messages were great, very motivational.”  (Chloe) “I think because it gradually started to feel a bit better, so I thought, oh, bloody hell this is actually working because you know how you get told some things and they’re going to work. Like telling someone to go on a diet but it doesn’t work. But I just felt, I don’t know, that’s why I say, I wonder if it was in my head because I felt better, I started to feel better. I mean the pain hasn’t 100% gone, don’t get me wrong, but it’s just better. It’s hard to explain. It’s good.” |
| Confidence to self-manage | (Harry) “I was just going to say that the exercises gave you the confidence to be able to maintain your regular lifestyle and that’s the main thing.”  (George) “I used to jump in the ute and I’d drive to the other end of the [shop], now I walk to the other end of the shop and back. So, it’s helped me with my mobility, and it’s helped me not only with my mobility but my self-confidence to be able to go, yeah, I can get up there all right and come down there.”  (Emily) “Well, as I said, being told that doing these things is not going to do more damage, and that was my initial thing. That was my biggest fear, yeah. So, I thought when I walked out of the doctor if that hurts don’t do it. And so, I came to an attitude where you pushed through a bit of pain”  (Charlie) “I was sick and tired of the pain, to be honest. I guess this whole journey for me has been, I got to a point where I was just sick and tired of being sick and tired of the pain, and I just wanted to do anything to solve it. And had you guys not come along I don’t think I would have got to a physio. So, there’s sort of all these different spin-off benefits to what the study’s done…  I couldn’t ride a pushbike at that time and that annoyed me. I just wanted to get the weight down and get on with it. And in the middle of all this I’ve lost 40 kilo as well.” |
| Encouraged active living | (Charlotte) “When my planters fasciitis got a bit better and I could actually sort of limp, I was going down to the gym in my building and cycling just to keep movement happening in my knee. That was what I picked up from the things [the program] that you do… I'm still doing that because like reading that, because when I thought it was bone on bone, you kind of think oh no, I'm grinding away at it and it’s going to make it worse. But after reading that, it was like keep moving, keep fluid happening through them and stuff.”  (Grace) “What it’s done is made me generally keep more active, walk wherever I can walk, instead of driving right up to the door of something I walk. It’s made me walk more briskly, I’ve made a conscious effort to do those things. But I do feel better, I feel better for all that.”  (Sophie) “I downloaded the log on that first day, and I never went back. I started to do little things and writing it, but I also use my step counter, and initially I can remember being quite shocked because I was only doing maybe 3-4,000 if I was lucky, steps a day. So, when I was away overseas, I was up to 14 or 15, and I don’t think I could’ve done that if I hadn’t done some of the strengthening beforehand.”  (Olivia) “I have been doing it for the last six months, it already became a habit. I have developed a habit of doing the exercise because I don't have pain anymore and I don't want to experience the same pain that I had before. So, I would really stick to this exercise and I will do it regularly.” |
| **Theme 5: Suggestions for real world application** | |
| Provided by a health professional preferred (General Practitioner or Physiotherapist) | (Chloe) “ …I’d been to the doctors and he would just say, later on down the track you’re going to probably need knee replacement and just…painkillers and all that and I like to walk so I found that hard. But once I started doing this, it’s just different and I wish the doctors would tell you, hey, go onto this website and read all this stuff, but they don’t….if you can get it through to the GPs and they can give it to people when they go in the first ever appointment and just say, well ok, check on this. If I’d known about it ten years ago, I don’t think I would have ended up as bad as I was.”  (George) “As part of that recovery process it would be ideal instead of just going, hey, you need to go to a physiotherapist for the next three months, or whatever, get … either doctors or physios to…”  (Amelia) “I could imagine a physio saying to somebody who’s coming in with some knee problems “Look at this programme. Look at this website and do those exercises as well as these other things I’m giving you. I’ll see you in a week or two weeks, and we’ll see how you’re going with it.” I could imagine that working quite well.” |
| Should be provided at subsidised or cheap out-of-pocket cost | (Liam) “A fee or have it funded through a Medicare levy, or through the Medicare programme. If it was recognised by Medicare, or in our case, because we’re on a pension, it would be covered by the government to a certain extent. If there was fee, and it was subsidised in some way, just like drugs were.”  (Joshua) “A lot of people that’ve got health insurance. health insurance could come to the party as well and pay some of it.”  (Grace) “I think people should pay for themselves, generally. So long as it wasn’t too expensive, because people won’t partake if it’s expensive, but I think that people have to have some motivation of their own and have to be willing to pay at least some small amount as long as it’s reasonable.” |
